# Community engagement for the rapid elimination of malaria: the case of Kayin State, Myanmar

**DOI:** 10.12688/wellcomeopenres.12051.1

**Published:** 2017-07-28

**Authors:** Ladda Kajeechiwa, May Myo Thwin, Suphak Nosten, Saw Win Tun, Daniel Parker, Lorenz von Seidlein, Decha Tangseefa, François Nosten, Phaik Yeong Cheah

**Affiliations:** 1 Shoklo Malaria Research Unit, Mahidol Oxford Tropical Medicine Research Unit, Mae Sot, Thailand; 2 Mahidol Oxford Tropical Medicine Research Unit, Faculty of Tropical Medicine, Mahidol University, Bangkok, Thailand; 3 Centre for Tropical Medicine and Global Health, Nuffield Department of Clinical Medicine, University of Oxford, Oxford, UK; 4 Faculty of Political Science, Thammasat University, Bangkok, Thailand; 5 The Ethox Centre, Nuffield Department of Population Health, University of Oxford, Oxford, UK

**Keywords:** community engagement, elimination, malaria, mass drug administration

## Abstract

Background: Currently, malaria elimination efforts are ongoing in several locations across Southeast Asia,  including in Kayin State (also known as Karen State), Myanmar . This paper describes the community engagement efforts for a pilot malaria elimination project, the challenges encountered and lessons learnt.

Methods: Between May 2013 and June 2015, a study on targeted malaria elimination (TME) that included mass drug administration was conducted in four villages (TPN, TOT, KNH, and HKT) of Kayin State. Community engagement efforts included workshops, meetings and house-to-house visits with community members.  Exhibitions related to malaria and fun activities were organized for children. In addition, we provided primary care, small individual incentives and village-level incentives. This paper is based on our analysis of data extracted from meeting minutes, field notes, feedback sessions among staff and with community members as well as our own reflections.

Results: Average participation across three rounds of MDA were 84.4%, 57.4%, 88.6% and 59.3% for TPN, TOT, KNH and HKT, respectively. Community engagement was fraught with practical challenges such as seasonal tasks of the villagers. There were challenges in explaining difficult concepts like drug resistance and submicroscopic infection. Another was understanding and navigating the politics of these villages, which are located in politically contested areas.  Managing expectations of villagers was difficult as they assumed that the community team must know everything related to health.

Conclusions: In the TME project, many different community engagement strategies were employed. We encountered many challenges which included logistical, scientific and political difficulties.  An approach that is tailored to the local population is key.

## Introduction

Although malaria-related morbidity and mortality has declined, the spread of drug resistant parasites in the Greater Mekong Subregion (GMS) poses serious challenges to prevention and control efforts ^1^. Multidrug resistant *Plasmodium falciparum*, the parasite that causes most of the deaths, is now established in the GMS ^2^.

To stop the spread of resistant *P.falciparum* strains, national malaria control programs have to focus on elimination rather than control. This includes establishing a malaria post in every village, in addition to other important interventions such as long lasting insecticide treated bednets ^3– 5^. Malaria posts are staffed by local villagers who have received training in diagnosis and treatment of malaria infections so that communities have ready and easy access to early diagnosis and treatment.

Mass drug administrations (MDA) of antimalarials are conducted to reduce the prevalence of asymptomatic infections, an important source of infections that are not addressed through passive case detection. MDA entails delivering a curative antimalarial dose to all individuals within a community, irrespective of malaria infection, to interrupt transmission. There is a direct, critical relationship between population coverage and outcome ^6^. Antimalarials need to be administered at the same time to the entire community to have an impact on malaria transmission. Effective community engagement before and throughout the MDA programme is indispensable to reach high coverage. Community engagement is also necessary to inform the potential participants of the benefits and risks of MDA, to encourage active participation and good adherence to the medication and to gain community trust.

With some notable exceptions ^7– 9^, there is a general lack of literature describing the various forms of engagement strategies in malaria elimination efforts, the people involved, how the activities are organized, and the strengths and challenges of these activities.

Currently, elimination efforts are ongoing in several locations across Southeast Asia, including in Kayin State (also known as Karen State), Myanmar, near the border with Thailand (ClinicalTrials.gov identifier: NCT01872702). This paper describes the community engagement efforts related to a Targeted Malaria Elimination (TME) project in four Kayin State villages, some of the challenges encountered and the lessons learnt. An inclusive definition of community engagement was adopted, ranging from one-to one-direct engagement targeted at key people such as village and religious leaders to the more indirect type of engagement with the wider community.

## Methods

### Setting

**Figure 1.**
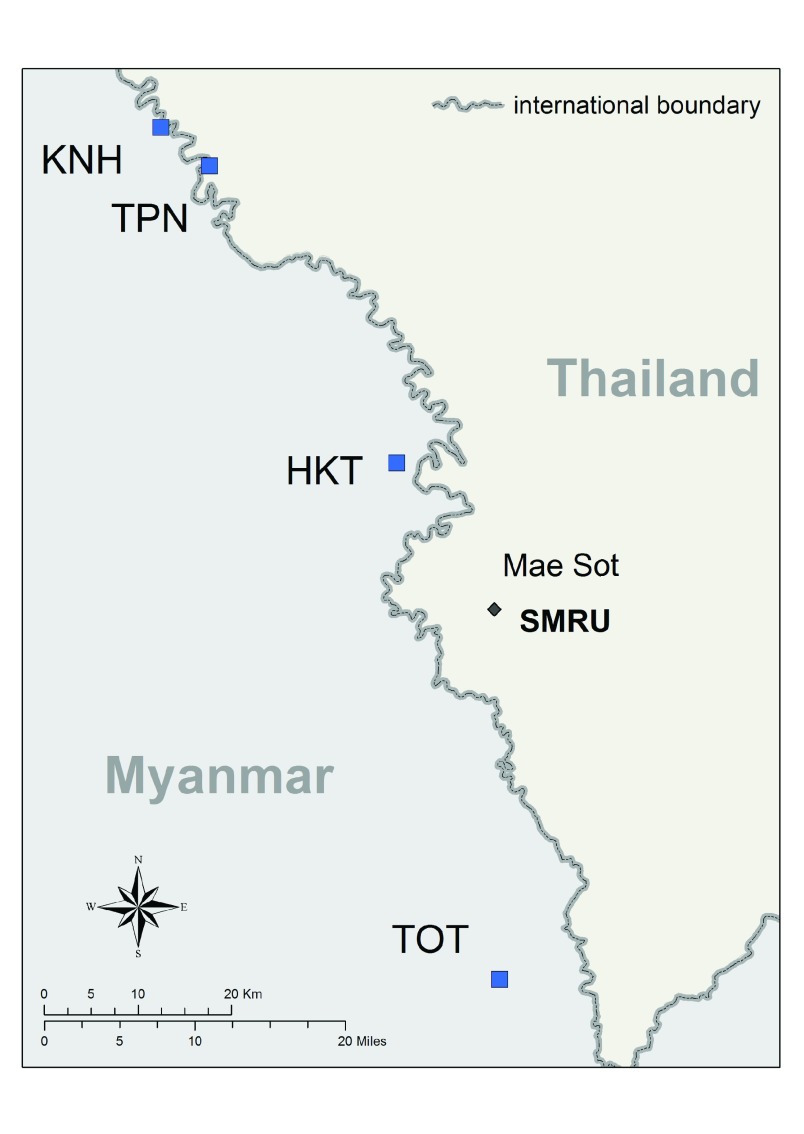
Map of the targeted malaria elimination villages.

Between May 2013 and June 2015, a study on TME that included MDA was conducted in four villages of Kayin State (KNH, TOT, TPN and HKT) ( Figure 1). In year one, two villages (TOT and KNH) underwent MDA and the other two served as ‘control’ villages (TPN and HKT). After nine months TPN and HKT underwent MDA, and TOT and KNH were ‘controls’. The villages are located in contested areas of Eastern Kayin State, with varying degrees of official government control and several influential armed groups. There have been varying levels of armed conflict in Kayin State since 1949 ^10^.

The distance between KNH, the northernmost study village and TOT, the southernmost is roughly 100km. The two northernmost villages are easiest to access from the Thailand side of the border. Access to TOT, can be difficult during the rainy season. The Shoklo Malaria Research Unit (SMRU) in Mae Sot, Thailand served as the operational headquarters for this TME project.

Most villagers were of the Karen ethnic nationality and S’gaw Karen was the most commonly spoken language. Eastern Pwo Karen and Burmese were also spoken in the villages. The majority of villagers were Buddhists, though some were Christians and many simultaneously practice animism.

Villages were selected based on a screening process using high volume ultrasensitive real time PCR (uPCR) ^11^, and a set of eligibility criteria which included high prevalence of submicroscopic malaria (greater than 30% positive for malaria of which 10% was falciparum malaria) and importantly, if the villagers and village leaders expressed willingness to participate in the MDA.

While the region has been in conflict for over six decades, various armed groups are currently engaging in peace discussions with the Myanmar government and armed conflicts are much less frequent. The transportation infrastructure in this part of the Kayin State has long been neglected, with very few all-weather roads. Current development projects are changing this landscape.

These factors have made the region a challenging setting for malaria elimination campaigns ^12^. Previous MDA campaigns for micro-filariasis control were unsuccessful in this setting.

TME -related activities consisted of the establishment of a malaria post in each study community, a census to establish the potentially eligible population. ‘Intervention’ villages received three rounds of MDAs, the first and last MDA were preceded and followed by prevalence surveys of the entire community using a highly sensitive uPCR ^11^. Malaria posts were established after the initial surveys, prior to MDA and were stocked with basic medical supplies and trained, paid staff. The three rounds of MDA were conducted one month apart, each round consisting of three daily doses of dihydroartemisinin-piperaquine combined with a single low dose primaquine on the first day of each round ^4^. All four villages during the TME study periods were asked to participate in 3-monthly surveys to detect submicroscopic malaria by uPCR. Between surveys, villagers were asked to approach their malaria posts if they had fever. Quantitative surveys to study reasons for non participation revealed that the main reason was inadequate understading of the rationale for MDA ^13^.

### Community engagement

The community engagement teams consisted of mainly local people (authors: LK, MMT, SN and SWT) and was led by a senior and respectable member of the Karen community (author: LK), supported by a central team (authors: DT and PYC). Having local and senior members from the Karen community was important so that engagement can be guided by adequate local knowledge and experience in the region, as well as access to target villages.

The following subsections describe the community engagement activities conducted for MDA in the KNH, TOT, TPN and HKT villages. These were based on meeting minutes, field notes, feedback sessions among staff and with community members as well as our own reflections.

***Workshops*.** Two-day training workshops were held with village volunteers and community leaders, including village leaders, village administrative staff, monks, and those responsible for health in the village. These groups consisted of 20 to 50 people. Topics covered in the workshop included those related to malaria, such as drug resistance and treatment for malaria. Villagers were encouraged to visit the malaria posts within 24 hours of experiencing fever or other symptoms that could potentially indicate malaria infection. There were discussions on MDA, its rationale and the related procedures, the reasons for uPCR testing, blood draws, and why participation of the entire population is important. The malaria lifecycle, how malaria is transmitted, the drugs used in MDA, potential adverse events related to the drugs and how to handle them were discussed. A quiz was conducted before and after the workshop to gauge understanding and to reinforce the message. Efforts were made to encourage questions as it is not in the local culture to ask questions in public meetings. These workshops were important as they addressed the fears and misconceptions of the villagers. In addition, village engagement strategies were discussed to specifically address cultural and religious aspects of engagement.

***Meetings*.** Meetings were held with groups of children, homemakers and youth groups. These meetings took place in village community halls, schools, temples and other places where groups of people routinely gather such as tea shops, farms and private homes.

In addition to these relatively formal meetings, the community engagement team regularly sought out ad hoc events with pre-existing social groups to talk about the TME project. One example for such spontaneous contact was a ladies social group at the TPN village who met at midday every day.

The community engagement activities were iterative. “Feedback meetings” were held by the team and village leaders with the goal of addressing queries from villagers about topics such as MDA-related rumours and adverse events. As the TME team consisted of healthcare providers, these small meetings also involved discussions about non-TME related everyday health problems, like seasonal illness and tiredness. Outsider groups were also asked to participate in the drug administrations and targeted with community engagement communications. These groups include armed forces, visitors, loggers or anyone who did not permanently stay in the village but visits it regularly.

House-to-house calls were made based on the census using house numbers. They were conducted seven days after MDA, and every two weeks for the entirety of the two-year project to take account of villager mobility and migrations, and to coincide with clinical case sessions at the malaria post. House calls were made by senior members of the team to people who declined to participate in the MDA in the evening when villagers had returned to their homes to talk about their concerns, worries and reasons why they would not or could not participate in the drug administration.

**Figure 2.**
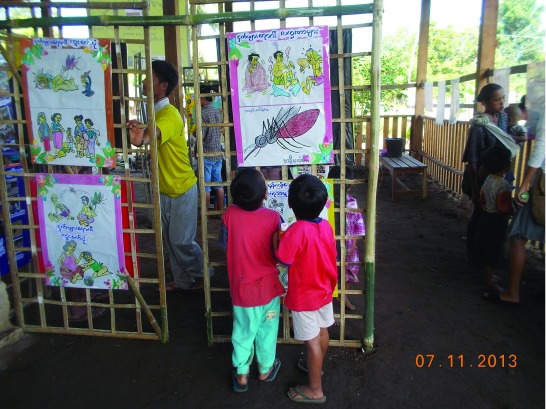
A children’s corner in a targeted malaria elimination village.

***Exhibitions*.** Maps, posters created by staff and displays of artwork that children created during engagement activities were exhibited in the space were villagers waited during the drug administration ( Figure 2). Topics covered included in these exhibits: impact of malaria, earlier spread of drug resistance to Africa such as chloroquine resistance; how malaria affects people, why uPCR is used versus rapid drug tests or microscopy, the MDA rationale, the Plasmodium lifecycle, how malaria transmits, drugs used and how blood samples are processed. Presentations were done using slide shows where possible, posters, drawings and discussions. In addition, locally available samples of antimalarial drugs, familiar to villagers were laid out and discussed.

***Activities for children and young people*.** Activities with children included colouring competitions, singing and acting with topics that were related to the malaria such as the Plasmodium life cycle, the blood volumes needed and the uPCR survey. Colouring was found to be very popular as it was difficult to get colour pencils in these villages. Singing, chanting and acting was also popular as this was entertaining and pleasurable for both children and their parents. There were also spontaneous sessions of games and activities for children unrelated to malaria.

***Incentives and ancillary care*.** While not really an incentive, community members did see the malaria posts as a benefit of being part of the project. Furthermore, water catchment and distribution systems and public latrines were built in each of the study villages. In meetings with the village leaders, water supply was identified as a priority by all four villages. These village-level benefits help build trust and ownership without the coercive element of individual incentives.

Health education unrelated to MDA was provided by the TME community engagement team to villagers at their request, for example family planning, nutrition and vaccination. Youth and healthcare staff benefited from health education and capacity building. Some young people were offered nursing and midwifery training and attachment at the Shoklo Malaria Research Unit so they can go back to work in their own villages more efficiently. Small gifts such as food bundles and household items (e.g. instant noodles, cooking oil, soap) were also given to villagers during MDA visits. No individual monetary incentives were provided.

***General rapport building activities*.** The community engagement team members embedded themselves in the community and engaged in general rapport building activities, frequently joining in village religious ceremonies such as the wrist tying ceremony, as well as rice planting and harvesting. The teams were hosted by villagers in their homes during the intensive MDA and survey days. Social activities with villagers allowed the team to learn more deeply about the realities of village life, seasonal work and obligations, and villagers’ priorities. This knowledge allowed us to plan community based events which were better attended when they did not conflict with villager commitments to their land, religious ceremonies or holidays.

Furthermore, these casual settings provided further opportunities to chat about the TME project, to hear villager comments and suggestions outside of formal settings or in front of the entire community. The makeup of the community engagement team allowed them to integrate themselves more deeply into normal villager life. Through these actions the team was not only able to gain deep insight into the communities, but they were also able to create a strong rapport with community members.

## Results

Average participation across three rounds of MDA were 84.4%, 57.4%, 88.6% and 59.3% for TPN, TOT, KNH and HKT, respectively ( Table 1).

This section describes the challenges and lessons learnt with regard to community engagement in TME.

**Table 1.** Participation rates by village and month of mass drug administration.

Village	TPN			TOT			KNH			HKT		
Month of MDA	1	2	3	1	2	3	1	2	3	1	2	3
Total participants taking 0 dose	22	54	60	171	236	210	17	27	39	281	356	278
(%)	6.4	16.6	19.8	29.1	43.4	37.2	5.6	7.7	10.9	38.0	44.1	32.8
Total participants taking an incomplete treatment (1 or 2 doses out of 3)	7	7	0	13	69	19	22	7	3	4	30	27
(%)	2.0	2.2	0.0	2.2	12.7	3.4	7.2	2.0	0.8	0.5	3.7	3.2
Total participants taking a complete 3-dose treatment	317	264	243	404	239	336	265	316	315	455	422	543
(%)	91.6	81.2	80.2	68.7	43.9	59.5	87.2	90.3	88.2	61.5	52.2	64.0
**Total population in the village** **during MDA round**	**346**	**325**	**303**	**588**	**544**	**565**	**304**	**350**	**357**	**740**	**808**	**848**

### Transportation and other logistical issues

Tak province, Thailand and Kayin state, Myanmar are separated by the river Moei (called Thaungyin River in Burmese language). Reaching the study villages requires crossing the river. KNH is easiest to access, while the most difficult to access village is TOT especially during the rainy season. Logistical issues include the absence of telecommunications facilities, absence of tarred roads, shops and facilities for the team to be based and frequent flashfloods. Finding accommodation for the research team was a challenge as was telecommunications. The study team lived with the villagers during intensive MDA and survey days. In villages without a phone network messages have to be hand delivered.

It is not possible to change the infrastructure of study villages hence more time than expected was needed to overcome logistical hurdles. It is essential to have a flexible schedule to suit the villagers’ needs.

### Timing and seasonality

In the early phase of the elimination project the team was not aware of the habits and seasonality tasks of the villagers. For example in the TOT village, the drug administration days clashed with the rice planting and mushroom collecting seasons, critical economic activities in the village.

It was not always possible to conduct the study activities at a time optimal for villagers. The timing of drug administrations and associated community engagement activities was dependent on many factors including the weather, local approvals and funding.

We found that it was important to involve schools and children in community engagement. The more children were involved in the engagement efforts, the better the overall participation in the MDA. For example, when we involved children in colouring contests and science drama, their friends and family members came to watch the activities, and were indirectly engaged. These activities were particularly well received during school holidays. In remote villages, activities for children such as colouring contests and science drama were popular as these activities were few and far between.

### Difficult concepts and rumours

Most villagers had limited or no formal education and had not travelled beyond the neighbouring villages which made it challenging to explain difficult concepts like drug resistance and submicroscopic infection. For example, early attempts to describe submicroscopic infections relied on a “tip of the iceberg” metaphor. Malaria parasites are normally detected using microscopy or rapid diagnostic tests. However, many infections in these communities existed at very low parasitemia levels, levels that were too low to be detected through normal detection methods. This means that only the “tip of the iceberg” was seen. This metaphor had little resonance with villagers who had never been to a cold country, heard of icebergs and much less seen a floating iceberg. The metaphor was changed to describe a large rock in a river, with only the top showing above the water – a scenario that most villagers well understood.

Engagement efforts were also frequently burdened by fears about potential adverse events following participation in the MDA. This was compounded by rumours related to a former filariasis MDA programme led by a non-governmental organisation which was a mixed success in this area.

Communicating difficult concepts such as drug resistance was challenging even when language competency was not an issue. Karen language has limitations with regard to scientific and technical terms. Concepts like evolution, mutation, MDA and asymptomatic malaria are complicated to translate, and usually require a phrasing that mixes Karen, English, Thai or Burmese. We had to find context-specific ways to convey the meanings of these concepts and this sometimes meant using different terms or phrases in different study villages. For example ‘malaria’ in Karen is ‘ta-nya-goh’ which translates to “fever with chilling”, but Karen people living in Thailand use specific term ‘pa-zo-su’ which means “poison from mosquito”. Another common misunderstanding is not having enough blood ‘thwee-t’pweh ba”, which is often thought by local community members to lead to low blood pressure and anaemia and is associated with giving blood (as in blood screenings for malaria).

We found that it was beneficial to check understanding such as introducing a quiz at the end of these sessions but courtesy suggests to announce such a quiz well in advance. Educational materials should be developed using variable approaches to achieve maximum versatility and to cater for sessions of various sizes, groups and locations. To minimize the problem of multiple translations and illiteracy, educational materials should have no text and consist of photos, hand drawn pictures and diagrams.

We learnt that the preparation, planning and delivery of community engagement requires time and resources. Only through adequate immersion in the culture and daily village life is it possible to build rapport with the villagers.

### Politics and power

Kayin State has seen clashes between government armed forces and arm groups for decades, therefore it was essential to understand which armed groups were in control of the area at the time of the project. Officially, the central government may wish to give the impression to be in control. The reality on the ground was more complex.

Equally important was to understand who was responsible for existing healthcare and who had influence in the villages. A good example was the TOT village, where there was divided leadership – with two major political and armed groups having a presence in the community. The overall community was not cohesive and the different political and armed groups supported their own healthcare initiatives, resulting in a confusing healthcare situation with different focus and agendas and high turnover of healthcare providers. In order for community engagement to be effective, the team had to engage with all of these groups and learn to effectively address issues. The malaria post had to be established in a way that all community members would take ownership.

### Expectations

Community engagement staff were perceived by villagers to be part of the TME medical team so the villagers expected that they must know everything related to health. Villagers often asked the team about everyday ailments, which the staff was unprepared to answer. We learnt that the community engagement team should include trained health care providers. Although their remit was not to provide long term healthcare, the provision of basic medications for minor ailments signals good will. In villages with inadequate medical care, the team has to be able to treat healthcare problems or refer villagers to appropriate health care providers.

### Unpredictability

There is no single set of operating procedures for community engagement. A major challenge was unpredictability which included misunderstandings by villagers and rumours about the objective of MDA. To be successful, community engagement teams must be flexible, mature and experienced, supportive of each other and resilient to work in difficult settings. Every team member of the TME project partook in community engagement one way or another.

## Discussion

### Community engagement activities

The activities conducted in relation to elimination, including the establishment of malaria posts and MDA, have similarities to campaigns described in the literature ^9^. Many reported health education campaigns and used a variety of strategies. For example in Nicaragua and Indonesia, large-scale health education campaigns on malaria were incorporated in the MDA programme ^7, 14^. In Tanzania, articles for the general public were written in two local newspapers ^15^. Our project provided small direct and indirect incentives to participants. MDA participants have been provided incentives that ranged from a lottery ticket and candy in Venezuela ^16^, to an advance for building a house in India ^17^. Similar to other engagement strategies, our project involved existing community structures. One example is Aneityum Island in Vanuatu, where village volunteers were responsible for drug distribution ^8^. The fact that the community engagment team was made up primarily of ethnic Karen people may also have influenced their acceptance in the communities, especially given the long history of conflict in Kayin State.

### Challenges and lessons learnt

Some of the challenges we encountered did not differ from those reported in literature, such difficulty in understanding complex medical concepts and the fact that the community expects the community engagement team to provide general healthcare ^18^. The lack of facilities such as telecommunication is also not unique to our setting ^19^. Others were more specific to our context such as political fragmentation, inaccessibility and high mobility.

We echo others who advocate that a high level of flexibility, adaptability, and motivation is required by the community engagement team ^20^. In addition, we think that some formative research and more creative methods such as science theatres could be used in addition to the more traditional methods of engagement ^21, 22^.

### Limitations and strengths

Our paper has several limitations. It is based on meeting minutes, field notes, feedback sessions and our own reflections rather than data systematically acquired. We also acknowledge that our work is not an extensive evaluation of our engagement activities. We were not able to assess the difference in the impact of the community engagement strategies that were employed. The MDA coverage was high in small villages (KNT and KNH) but not in the larger ones (TOT and TPN), and it was not possible to determine a direct relationship between community engagement and population coverage.

Our previous questionnaire survey study on community perceptions on MDA revealed that an important reason for non-particpation an inadequate understanding of the intervention ^13^. We think that community engagement plays an important role in facilitating this understanding. We have learnt important lessons about community engagement from our rich field experience that may be applicable to similar MDA programmes.

## Conclusions

In the TME project, many different community engagement strategies were employed. We encountered many logistical, scientific and political challenges. We think that an approach that is tailored to the local population, meeting their local needs and understanding their local problems, is most effective not only to improve coverage and maximise success of the MDA programme, but also to promote goodwill and trust. Such a program will inherently need to draw on local expertise and the ability of the community engagement team to be able to adapt to regular feedback.

## Data availability

The data referenced by this article are under copyright with the following copyright statement: Copyright: © 2017 Kajeechiwa L et al.

Due to ethical and security considerations, the data that supports the findings in this study can be accessed only through the Data Access Committee at Mahidol Oxford Tropical Medicine Research Unit (MORU). The data sharing policy can be found here: http://www.tropmedres.ac/data-sharing. The application form for datasets under the custodianship of MORU Tropical Network can be found as a Supplementary File.

## Ethical approval

This study is has been approved by the Oxford Tropical Research Ethics Committee (ref: 1017-13) and the Tak Province Community Ethics Advisory Board.

## Supplementary material

**Supplementary File 1: Application form for datasets under the custodianship of Mahidol Oxford Tropical Medicine Research Unit (MORU) Tropical Network.**
