# Supplementary material for: Community engagement for the rapid elimination of malaria: the case of Kayin State, Myanmar
Source: Wellcome Open Res. 2017 Jul 28;2:59. [Version 1] doi: 10.12688/wellcomeopenres.12051.1 (PMC5580421; doi:10.12688/wellcomeopenres.12051.1)
Supplement: Supplementary file 1 [file wellcomeopenres-2-13037-s0000.tgz › 15d0c8c2-e618-45cf-bd7d-f104da59a571.docx]

**Application Form for Datasets under the Custodianship of Mahidol Oxford Tropical Medicine Research Unit (MORU) Tropical Network**

| **1. APPLICANT DETAILS** | | | | | | |
| --- | --- | --- | --- | --- | --- | --- |
| **Name of applicant** |  | | | | | |
| **Institution name and address** |  | | | | | |
| **Phone** |  | **Email** |  | | | |
| **Name of applicant’s supervisor/ manager/ head of department (delete as appropriate)** |  | | | | | |
| **Phone** |  | **Email** |  | | | |
| **2. DETAILS OF PROJECT** | | | | | | |
| **Title of Project** |  | | | **Start Date** | |  |
|  |  |  |  | **End Date** | |  |
| **3. CO-APPLICANTS INCLUDING MORU COLLABORATORS (if applicable)** | | | | | | |
| **Name** | **Role** | | | | **Institution** | |
|  |  | | | |  | |
|  |  | | | |  | |
| **Anticipated users or user groups other the above individuals e.g. data management team of applicant’s institution** |  | | | | | |
| **4. BRIEF DESCRIPTION OF DATA REQUESTED** | | | | | | |
|  | | | | | | |
| **5. OBJECTIVES OF PROJECT** | | | | | | |
|  | | | | | | |
| **6. BRIEF DESCRIPTION OF ANALYSIS PLANNED** | | | | | | |
|  | | | | | | |
| **7.POTENTIAL ETHICAL ISSUES INCLUDING RISKS e.g. stigmatisation or breaches of privacy** | | | | | | |
|  | | | | | | |
| **8.POTENTIAL BENEFITS OF THE STUDY including to participant communities, scientific capacity building or health policy** | | | | | | |
|  | | | | | | |
| **9. PLANNED OUTPUTS including publications** | | | | | | |
|  | | | | | | |

**Signature of applicant: _____________ DATE: ______**

**Signature of applicant’s supervisor/ manager/ head of department: _____________ DATE: _______**

Note: Additional information may be requested to support the application. We are not charging for data access but the applicant may be required to cover the cost of preparing the data for sharing. Primary contact: phaikyeong@tropmedres.ac
