# Supplementary material for: Community engagement for the rapid elimination of malaria: the case of Kayin State, Myanmar
Source: Wellcome Open Res. 2017 Jul 28;2:59. [Version 1] doi: 10.12688/wellcomeopenres.12051.1 (PMC5580421; doi:10.12688/wellcomeopenres.12051.1)
Supplement: Peer review history

# Peer review: Referee response for version 1

**DOI:** 10.21956/wellcomeopenres.13037.r24654

**Published:** 2017-08-29

**Authors:** James V. Lavery

**Affiliations:** 1 Hubert Department of Global Health, Rollins School of Public Health, Center for Ethics, Emory University, Atlanta, GA, USA

**General Comments**

1. The paper is significant for several reasons. First, it provides a solid example of how to report the process and outcomes of community engagement (CE) strategies in global health and global development. Despite an improved appreciation of community engagement overall, there are still very few examples of competent reports of CE strategies in the literature. This has limited the empirical footprint of CE and hampered important learning for funders, implementation partners, researchers and a wide range of review panels. Second, although only partially successful in this case, the paper makes an important effort to link CE activities with relevant outcomes of interest - i.e., participation rates in MDA. “Partial success”, in this case, has a great deal to do with the lack of well-developed models or comparators, so although there are points that could certainly be elaborated and clarified, the paper provides a concrete example that can be constructively critiqued and built upon, which is a fundamentally important contribution to the literature in itself. Third, malaria elimination has become a dominant focus in malaria research and intervention. Funders and implementation partners have recognized the importance of CE as a key element of MDA and other elimination strategies, but still struggle to understand the unique value proposition of CE and how best to integrate it into their investments. This paper contributes to a growing body of knowledge that will help to address these important upstream challenges in global health. 
2. A key strength of the paper lies in how explicitly it attempts to account for the elements of the CE process. There are important gaps (see below), but overall the paper provides a vivid and useful account of what was done, if slightly less detail about the rationales, outcomes and implications.
3. A weakness of the paper, as suggested above, is the relative lack of attention to the rationales for the various decisions about CE mechanisms - i.e., why, precisely, were the various approaches selected - and to the outcomes and implications of each of these decisions/activities/mechanisms. One of the important gaps in the CE literature is program theory, i.e., coherent accounts of configurations of context + mechanisms + outcomes that can provide us with a better understanding of how CE actually works in any specific context of application. The paper succeeds, in my view, by virtue of providing a relatively rich description of the CE activities, but further elaboration on the rationales, outcomes and implications, in addition to description of some of the activities/mechanisms, would have made the paper much stronger. (See detailed comments, below)
4. Another weakness of the paper, which might be easily remedied, is that the discussion section is very limited and does not deal very effectively with the findings reported in the paper. If there is any scope of revision of the paper, I would suggest that some of the detailed comments, below, might provide a useful guide for what informed readers might find most helpful in terms of elaboration. Since each of these points arises from a comment on something in the paper it should be feasible, and not onerous, for the authors to simply expand the discussion section of the paper to share some of their thoughts and reflections more explicitly on these, and perhaps other, points.

 

**Specific Comments** 

1. **Page 3, column 1, 5 ^th^ paragraph: “An inclusive definition of community engagement was adopted…”**
  This is a good example of how the framing and reporting of CE is limited primarily to CE *mechanisms* or *activities*. It would have been extremely helpful, especially at the outset of the paper, to have some elaboration of the goals and rationales lying behind these definitions. This would also have provided some conceptual space for the authors to report on other relevant observations.
2. **Page 4, column 1, 2 ^nd^ sentence: “Previous MDA campaigns for micro-filariasis control were unsuccessful in this setting.”**
  Why? Were the failures the result of poor design? Poor execution? Or contextual features of the region, etc.?
3. **Page 4, column 1, 2 ^nd^ paragraph, final sentence: “Quantitative surveys to study reasons for non-participation revealed that the main reason was inadequate understanding of the rationale for MDA** *.”*
  What are the implications of this finding? Does CE need to focus more on the provision of information? More on measuring understanding? Etc.
4. **Page 4, column 1, last line: “…fears and misconceptions…”**
  What were these fears and misconceptions? How were they addressed? What are the broader lessons for CE?
5. **Page 4, column 2, 3 ^rd^ full paragraph, 1 ^st^ sentence: “The community engagement activities were iterative.”**
  In what sense? What insights were derived from the activities? How were these insights utilized to revise/refine approaches? Etc.
6. **Page 4, column 2, 4 ^th^ full paragraph, last sentence: “…concerns, worries and reasons why they would not or could not participate in the MDA.”**
  What were these concerns, worries and reasons? What are the implications for CE? Are we looking at the right outcome measures? Etc.
7. **Page 4, column 2, 5 ^th^ full paragraph, first sentence: “Exhibitions”**
  What is the intended purpose of these exhibitions? The paper describes them, but does not link them back into an explicit goal(s) or rationale(s) or outcome(s).
8. **Page 4, 2 ^nd^ column, last sentence: “Singing, chanting and acting was (sic) also popular as this (sic) was entertaining and pleasurable for both children and their parents.**
  Again, what was the intended purpose of these activities? How should the pleasure of children and parents be interpreted in term of the operating logic of the CE strategy?
9. **Page 5, 1 ^st^ full paragraph, last sentence: “This knowledge allowed us to plan community based events which were better attended when they did not conflict with villager commitments to their land, religious ceremonies or holidays.”**
  Again, what were these insights? How, specifically, were they addressed—i.e., what kinds of revisions and refinements to the design of the CE strategy did these insights lead to?
10. **Page 6, 1 ^st^ paragraph, second last sentence: “The study team lived with the villagers during intensive MDA and survey days.”**
  Although I understand this point both from a cultural and pragmatic perspective, given the locations of the study, this seems like quite a profound point in terms of our understanding of “engagement”. What did the workers learn? What were the hosts experiences? Even if these aren’t reported in a comprehensive way, are their key insights or lessons that might help readers gain better understanding of CE, especially the relational aspects?
11. **Page 6, column 2, 4 ^th^ full paragraph, last 2 sentences: “Officially, the central government may wish to give the impression to be in control (sic). The reality on the ground was more complex?**
  In what way(s)? What were the implications for the design and conduct of the CE strategy?
12. **Page 6, column 2, 5 ^th^ full paragraph, first sentence: “Equally important was to understand who was responsible for existing healthcare and who had influence in the villages?**
  Why?
13. **Page 6, column 2, 5 ^th^ full paragraph, last sentence: “The malaria post had to be established in a way that all community members would take ownership.”**
  “Ownership is a commonly used term. Unfortunately, the possible meaning(s) is not self-evident here and seems quite important in terms of the overall goals of the CE strategy.
14. **Page 7, first paragraph, line 3: “…signals good will…”**
  What are the implications of signaling good will here? Is this an explicit goal of CE, is it an instrumental goal? Or an end itself for CE?
15. **Page 7, column 1, 4 ^th^ full paragraph, last sentence: “…we think that some formative research and more creative methods such as science theatres could be used in addition to the more traditional methods of engagement.”**
  Why, what goals would these serve? What outcomes would they produce?

 

**Recommendations**

 As I hope my specific comments make clear, there are some missed opportunities in the paper to provide some elaboration on several quite important issues. However, given the current state of the literature I think the paper represents an solid and important attempt to build our understanding of CE, especially in such an important global health context—MDAs in malaria elimination—and therefore I would recommend indexing the paper.

 In terms of what revisions are necessary, I think this sits somewhere between minor revisions and major revisions. One approach, as I have outlined above, would be for the authors to pick up on some, or all, of my detailed comments, above and incorporate them into a more robust discussion section. As it stands, the discussion is extremely lean and focuses primarily on other work. Some or most of this other work would make more sense as *background* and would make way for the authors to provide more detailed reflection on their findings and experiences. There are probably 4-5 main themes in my specific comments and these might offer a useful framing for the kinds of insights and elaboration that would have most value for an informed reader.

I have read this submission. I believe that I have an appropriate level of expertise to confirm that it is of an acceptable scientific standard.

# Peer review: Referee response for version 1

**DOI:** 10.21956/wellcomeopenres.13037.r24651

**Published:** 2017-08-10

**Authors:** Kate Gooding

**Affiliations:** 1 Malawi-Liverpool-Wellcome Trust Clinical Research Programme, Blantyre, Malawi; 2 Liverpool School of Tropical Medicine, Liverpool, UK

This article provides a useful and interesting account of community engagement for a malaria elimination programme involving mass drug administration. The article describes the community engagement activities, the challenges and some of the steps taken to overcome these challenges. Honest reports of community engagement are a valuable resource for other practitioners. This article reports engagement in a particularly challenging context with geographical, social and political difficulties, so highlights some of the complications in engagement. The detailed account of engagement activities also provides useful ideas and learning for other engagement practitioners (e.g. the use of a quiz to check understanding), and there are nice examples of making engagement culturally appropriate (e.g. using of a rock rather than iceberg metaphor to describe malaria levels). I also welcome inclusion of an article that is based on team reflections rather than additional data collection; reflection is an essential part of good research and engagement practice, and useful lessons can be missed without openness to this kind of article.

 A few areas might be suggested to further deepen the analysis:

 It would be interesting to learn more about variations between the villages, and about whether particular engagement strategies were more effective in some contexts than others. The authors note that it was not possible to determine a relationship between engagement and coverage or to assess the impact of different activities. However, additional descriptions of any variation in activities between sites and reasons for this might be helpful, together with reflections on potential reasons for variations in coverage and effectiveness of CE. For example, the surveys suggested that people did not participate due to inadequate understanding of MDA, indicating limitations of the CE. It would be interesting to reflect on the extent to which this involves limited implementation of CE (e.g. due to geographical barriers), or ineffectiveness of CE that was conducted (e.g. due to problems with translation), and how these constrains varied between villages. Some aspects of variation are indicated, but could perhaps be brought together more explicitly.

 The authors and engagement team are described as local. Local is a word that can be interpreted in different ways, so it would be interesting to reflect on their position and to give more detail on their relationship to communities. Were they local in the sense of coming from these communities, or from the region, and were they considered as local by the communities? Relationships may be affected by similarity or difference in terms of social status, education, etc., as well as residence or place of birth.

 Another area for further reflection relates to the idea of engagement as a two-way process that involves seeking feedback from communities. The focus of engagement appears to have been on informing communities about MDA to promote high coverage. It may be useful to say more about any aims or strategies around eliciting community feedback on the intervention, and the effects of such strategies.

 While the article is well written, occasionally suggestions are made about what approach should be taken when it is not fully clear what was actually done or what challenges arose (e.g. in relation to education materials and unpredictability in the results section). It may might be useful to clearly separate lessons and recommendations from descriptions of what happened. It may also be useful to summarise key lessons in a box in the discussion or conclusions – there are valuable suggestions and a box would help to ensure that lessons are seen by busy readers. To support learning, it would also be helpful to have a little more explanation and detail on some of the points mentioned in the results, for example, establishing a malaria post in a way that enabled ownership – ownership is a common requirement and challenge, so it would be interesting to know how it was achieved.

 Some lessons are fairly standard recommendations for engagement with development interventions and widely advocated, such as the need for adequate time and flexibility. Do the authors have any reflections on why these essential resources and features of engagement approaches are not always followed, or on potential constraints to adoption of this advice? What systems or approaches can help to ensure, for example, that teams are flexible?

 Consideration of other literature in the discussion section notes other CE programmes that have followed a similar approach. It would be useful to extend consideration of the literature to see whether other articles have reflected on the strengths and weaknesses of the different CE activities (such as individual or community compensation), to draw out wider lessons on advantages and challenges.

 These comments do not affect the value or conclusions of the article; they are areas of further interest for the authors to consider rather than requirements for re-writing. I would like to thank the authors for the opportunity to review this interesting article and for their contribution to the learning on engagement.

I have read this submission. I believe that I have an appropriate level of expertise to confirm that it is of an acceptable scientific standard.
